# Loss of Akt3 enhances antiviral immunity while disrupting corneal homeostasis during ocular HSV-1 infection

**DOI:** 10.3389/fimmu.2026.1847233

**Published:** 2026-08-12

**Authors:** Rashmi Kadam, Chandrashekhar D. Patil, Hemant Borase, Rakesh Rahangdale, Ipsita Volety, Soromidayo Akinsiku, Deepak Shukla

**Affiliations:** 1 Department of Ophthalmology and Visual Sciences, College of Medicine, University of Illinois Chicago, Chicago, IL, United States; 2 Department of Biochemistry and Molecular Genetics, College of Medicine, University of Illinois Chicago, Chicago, IL, United States; 3 Department of Immunology and Microbiology, College of Medicine, University of Illinois Chicago, Chicago, IL, United States; 4 Department of Pathology, College of Medicine, University of Illinois Chicago, Chicago, IL, United States

**Keywords:** Akt3, eye infection, HSV - herpes simplex virus, immune response, neutrophils

## Abstract

**Background:**

Akt1 and Akt2 isoforms reveal distinct immune-regulatory roles in HSV-1 pathogenesis. Akt3 is a developmentally important protein, and mutations in Akt3 gene are associated with a wide spectrum of developmental disorders, including extreme megalencephaly; however, its role in corneal homeostasis and pathologies remains poorly understood. Here we investigated the role of Akt3 in HSV-1 infection.

**Methods:**

*In vivo* HSV-1 infection was performed using WT, Akt3 homozygous, and heterozygous mutant mice via corneal scarification to assess disease outcomes. Viral replication was quantified by plaque assays from ocular samples at defined time points. Mice were subjected to ex vivo and *in vitro* analyses, including flow cytometry, ELISA, and neutralization assays, to comprehensively evaluate innate and adaptive immune responses.

**Results:**

In this study, we report the significance of the Akt3 isoform in HSV-1 viral pathogenesis. Loss of Akt3 at baseline results in spontaneous corneal opacity, suggesting it is a critical regulator of ocular homeostasis. Despite this baseline compromise, Akt3 deficient mice appear to be better protected against HSV-1 infection, showing lower titers and better survivability. Loss of Akt3 plays a unique role in regulating type 1 interferon production necessary for combating viral infection, along with demonstrating significantly higher serum neutralizing capacity compared to WT controls.

**Conclusion:**

Akt3 deficiency reconfigures the innate-adaptive interface acting as a negative modulator of the antiviral response during HSV-1 infection.

## Introduction

The Akt protein family or protein kinase B compromises three isoforms; Akt1, Akt2, and Akt3, which are encoded by distinct genes but share significant structural homology. These isoforms serve as master regulators of cellular homeostasis, governing essential processes like glucose metabolism, cell growth, and survival (1). While Akt3 was thought to be restricted to brain and testis in murine models, a significant knowledge gap remains regarding its expression in peripheral organs and its functional relevance in those tissues (1, 2). Notably, there is currently no clear understanding of its role in maintaining corneal clarity or in modulating viral pathogenesis in the eye, including host responses to ocular HSV-1 infection.

The modulation of Akt-mediated signaling has profound implications for disease outcomes, particularly during viral infections (3–5). Herpes simplex virus type 1 actively engages PI3K/Akt signaling to promote viral entry and replication. Binding of HSV-1 glycoproteins to the cell surface triggers rapid Akt phosphorylation, which facilitates integrin signaling, intracellular calcium mobilization, and focal adhesion kinase activation all essential for efficient viral entry. Additionally, Akt signaling modulates both innate and adaptive immune responses to viral infection (6, 7). Our group has previously shown the non-redundant roles of Akt1 and Akt2 in HSV-1 pathogenesis, demonstrating that the presence of Akt1 or the absence of Akt2 is critical for host antiviral immunity (8). However, the specific contribution of Akt3 within host-pathogen interactions remains unclear.

Herpes simplex type 1 (HSV-1) is a neuroinvasive pathogen that initially targets epithelial barriers such as oral or ocular mucosa (9, 10). Upon viral entry, host cells mount complex defense responses including triggering a cascade of cytokines and chemokines to recruit immune effector cells and establishing a robust antiviral state coordinated across several cell types through the production of host molecules like IFNβ (11). The outcome of primary ocular HSV-1 infection, whether the virus is rapidly cleared or establishes productive infection, depends critically on the integrity of the corneal epithelial barrier and the coordinated innate and adaptive immune responses mounted against the pathogen (12, 13). Despite the importance of corneal homeostasis in controlling infection, the molecular regulators of corneal epithelial function during viral infection remain incompletely understood (14).

Here, we report a dual role for Akt3 in the eye: sustaining corneal transparency and regulating the host antiviral response. We show that the damaged corneal environment in Akt3-deficient mice acts as a catalyst for a more robust immune state, characterized by elevated IFNβ and potent humoral immunity. These results challenge the assumption that developmental/mechanical defects increase infection risk, providing new insights into how Akt3 balances tissue integrity with systemic defense.

## Materials and methods

### Animals

C57BL/6 mice were obtained from Jackson Laboratory (JAX) stock 000664. All the models used in this study are generated in a C57BL/6 background; therefore, C57BL/6 mice have been used as the WT control model. Akt3^-/+^ and Akt3^-/-^ models were kindly shared by Prof. Nissim Hay (University of Illinois Chicago, Chicago). All experiments were performed and housed in a BSL2 rodent facility in the Biologic Resources Laboratory at the University of Illinois, Chicago, with a standard 12 h light, 12 h dark cycle. Ambient temperatures at this facility are maintained between 20 and 26 °C and relative humidity between 45% and 65%. All protocols have been reviewed and certified by the animal care committee of the University of Illinois Chicago (protocol # 23-027). Male and female mice (8–12 weeks of age) were included in the baseline corneal phenotype analysis and in the HSV-1 infection experiments to minimize variability. To ensure consistency across genotypes and reduce variability due to baseline ocular abnormalities, eyes were selected for infection based on clinical appearance prior to inoculation. Only eyes without severe pre-existing damage were included. In Akt3 heterozygous mice, when one eye exhibited opacity, structural damage, or spontaneous closure, the contralateral eye was selected for infection. Animals with bilateral ocular abnormalities were excluded from the study. In Akt3 homozygous knockout mice, unilateral abnormalities were infrequent; when present, the unaffected eye was selected for infection. In wild-type mice, the right eye was consistently used in accordance with the standard experimental protocol. This selection strategy was applied uniformly across all experimental groups to ensure comparable baseline conditions at the time of infection.

### *In vivo* HSV-1 infection

HSV-1 infection was performed by the corneal scarification method. At the time of infection, mice were anesthetized with an intraperitoneal injection of ketamine (100 mg/kg)-xylazine (10 mg/kg) mixture, and a drop of topical anesthetic, Proparacaine hydrochloride 0.5%, was applied to the ocular surface prior to epithelial debridement. Their corneas were scarified in a 3 x 3 grid using a 30-gauge needle, followed by the application of HSV-1 McKrae at 10^6^ PFU. On days 1, 2 and 4 post-infection, eye wash samples were collected to quantify HSV-1 replication through plaque assay. Acute weight loss (>20%) and hunching of the animal were considered as clinical endpoints. Mice were euthanized by gradual-fill CO_2_ inhalation using a displacement rate of 30-70% of the chamber volume per minute, followed by a secondary physical method to confirm death, in accordance with institutional IACUC guidelines and the AVMA Guidelines for the Euthanasia of Animals. The corneal disease scoring criteria are as follows: 0 = healthy, 1 = opaque, and 2 = closed eye. All corneal assessments were performed in a blinded manner to avoid bias.

### Isolation of lymphoid organs and analysis by flow cytometry

We isolated cervical lymph nodes from WT and transgenic animals (Akt3^-/+^ and Akt3^-/-^). Immune cells were harvested by crushing cLN and spleen using the 5-cc syringe. Piston/plunger was removed, and the flat end of the plunger was used to mince the spleen and cLN by crushing in a gentle circular motion. Isolated cells were stained with respective antibodies at a 1:200 dilution (**Table 1**). Live/Dead dye was used according to the manufacturer’s recommendations. Samples were acquired on Cytek Aurora multispectral flow cytometer and analysis was conducted on FlowJo 10.10 software.

**Table 1:** List of reagents.

Reagent or resource	Source	Identifier
Antibodies		
Anti-mouse CD45-BV510 (clone 30-F11)	BioLegend	Cat#103138; RRID: AB_2563061
Zombie NIR dye	BioLegend	Cat#1423105
Anti-mouse CD3-AF700 (clone 17A2)	BioLegend	Cat#100216; RRID: AB_493697
Anti-mouse CD3-APC (clone 17A2)	BioLegend	Cat#100236 RRID: AB_2561456
Anti-mouse CD4-PE-CY7 (clone RM4-5)	BioLegend	Cat#100528; RRID: AB_312729
Anti-mouse CD8a-PerCP/Cy5.5 (clone 53-6.7)	BioLegend	Cat#100734; RRID: AB_2075238
Anti-mouse CD11c-BV785 (clone N418)	BioLegend	Cat#117335; RRID: AB_11219204
Anti-mouse NK1.1- FITC (S17016D)	Biolegend	Cat#156507; RRID: AB_2876526
Anti-mouse CD11b-BV711 (clone M1/70)	BioLegend	Cat#101242; RRID: AB_2563310
Anti-mouse Ly6G-AF647 (clone 1A8)	BioLegend	Cat#;127610 RRID: AB_1134159
Anti-mouse Ly6C- BV421 (clone HK1.4)	BioLegend	Cat#; 128032 RRID: AB_2562178
Anti-mouse F4/80-PE (clone BM8)	BioLegend	Cat#123110; RRID: AB_893486
Anti-mouse CD19-Spark Blue 574 (clone 6D5)	BioLegend	Cat# 115582, RRID: AB_2904287
Anti-mouse MHC II-PerCP/Fire 806 (clone M5/114.15.2)	BioLegend	Cat#107673 RRID: AB_2941380
Anti-mouse MHC I-APC Fire 750 (clone 28-8-6)	BioLegend	Cat#114617 RRID: AB_2750197
Anti-mouse XCR1-BV605 (clone ZET)	BioLegend	Cat#148222; RRID: AB_2927815

We used the gating strategy as follow: CD4 T cells = Live^+^CD45^+^CD3^+^CD4^+^; Activated CD4^+^ T cells = Live^+^CD45^+^CD3^+^CD4^+^25^+^; CD8 T cells = Live^+^CD45^+^CD3^+^CD8^+^; B Cells = Live^+^CD45^+^CD19^+^; Macrophages = Live^+^CD45^+^CD3^-^F4/80^+^CD11b^+^; Total DCs= Live^+^CD45^+^CD3^-^F4/80^-^CD11c^+^; NK Cells = Live^+^CD45^+^CD3^-^NK1.1; Monocytes= Live^+^CD45^+^CD3^-^F4/80^+^CD11b^+^Ly6C^+^; Neutrophils= Live^+^CD45^+^CD3^-^F4/80^-^CD11b^+^Ly6G^+^.

### Plaque assay

The Vero cells were seeded in a 24-well tissue culture plate to form a monolayer. The monolayer was infected with respective dilutions of eye wash in Opti-MEM. At 2 hpi, the dilutions of eye washes were aspirated and replaced by a complete medium (DMEM with 10% FBS and 1% PenStrep) containing 0.5% methylcellulose (MC-DMEM). The cells were incubated at 37 °C in 5% CO_2_ for 72 h. After incubation, the cells were fixed by adding 100% methanol. After 30 min of fixation, the medium was removed, and the cells were stained with a crystal violet solution. The number of plaques was counted visually, and statistical analysis was performed.

### Serum collection

Post euthanasia blood was collected using a BD Syringe with Micro-Fine Needle (28G, model #329461) from the heart of murine animals in polystyrene 1.5 ml Eppendorf tubes. Samples were incubated at RT for 2 h, followed by centrifugation at 3000 x g for 5 mins at 4 °C. Serum was collected from the top layer and stored at -20 °C until further analysis.

### ELISA

ELISA was performed according to the manufacturer’s protocol for measuring immunoglobulin in murine eye washes and serum. (LEGENDplex™ Mouse Anti-Virus Response Panel (13-plex) with V-bottom Plate, catalog no. 740622, Biolegend).

### Neutralization assay

Serial dilutions were prepared from serum samples in Opti-MEM in a 48-well plate. 100 virus particles of the McKrae strain were used to assess the neutralizing response of serum antibodies. In equal volume, 100 virus particles were added to the pre-diluted serum samples and incubated for 2 h at 37 °C. A virus with no serum was used as a control. Co-incubated serum -virus was transferred to 24-well plates containing monolayers of VERO cells, followed by 2 h incubation at 37 °C. After 2 h incubation, the media was aspirated and replaced with a 0.5% MC-DMEM solution. The cells were incubated at 37 °C, 5% CO_2_, for 72 h. After incubation, the cells were fixed by adding 100% methanol at RT. After 30 min of fixation, the medium was removed, and the cells were stained with a crystal violet solution. The number of plaques was counted visually, and statistical analysis was performed.

### Quantification and statistical analysis

Flow cytometry analyses were performed using FlowJo software (version 10.8.1, win64). Statistical comparisons were performed using GraphPad Prism software (version 10.2.3) and ImageJ (version 1.8.0). Unpaired two-tailed Student’s *t*-test or one-way ANOVA, Tukey’s *post hoc* analysis was performed to determine statistical significance between two samples or for multiple comparisons, respectively. All data are from at least two independent experiments and presented as means ± SD. The significance was when *p* values were <0.05 (represented as *p < 0.05, **p < 0.01, ***p < 0.001, or not significant (NS)).

## Results

### Akt3 deficiency leads to corneal opacity

**Figure 1:**
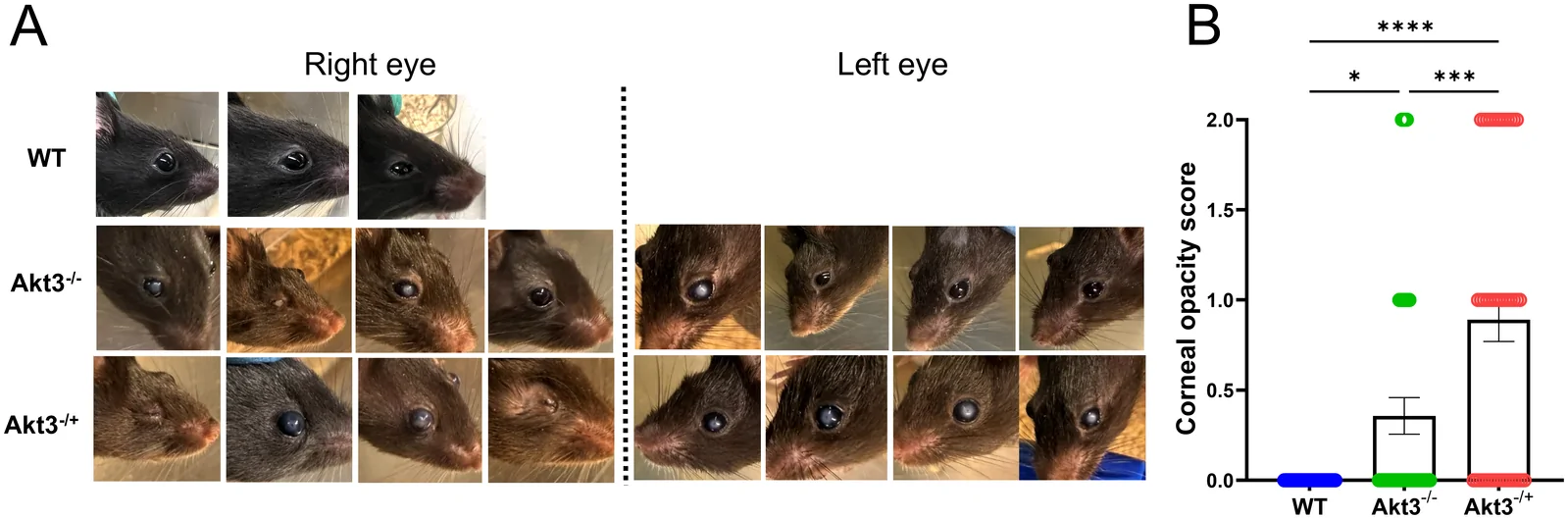
Akt3 deficiency is associated with corneal opacity. **(A)** Representative baseline images of eyes from WT, Akt3^-/-^, and Akt3^-/+^ mice. The images shown are representative of the animals analyzed in each group (WT, *n* = 3; Akt3^-/-^, *n* = 4; Akt3^-/+^, *n* = 4). **(B)** Corneal pathology scores for WT (*n* = 21), Akt3^-/-^ (*n* = 21), and Akt3^-/+^ (*n* = 23) mouse eyes. Both eyes from male (*n* = 9-14) and female (*n* = 7-12) mice were evaluated. Corneal disease was scored as follows: 0 = healthy, 1 = corneal opacity, and 2 =complete eye closure. Data are presented as mean ± SEM. Statistical analysis was performed using one-way ANOVA. Significance is indicated as ∗p ≤ 0.05, ∗∗∗p ≤ 0.001, and ∗∗∗∗p ≤ 0.0001.

In the current study, we challenged wildtype (WT), Akt heterozygous (Akt3^-/+^) and Akt homozygous (Akt3^-/-^) mice with HSV-1 to investigate Akt3’s role in viral pathogenesis. We identified spontaneous corneal opacity in mice lacking Akt3, which was surprising, given that previous reports suggest that Akt3’s function is limited to the brain in murine models. Representative images (**Figure 1A**) show that while both Akt3^-/-^ and Akt3^-/+^ mice develop corneal opacity, the Akt3^-/+^ genotype often presents with more severe pathology, including corneal damage leading to eyelid closure. This phenotype showed no lateral preference, affecting either eye randomly. Quantifying these observations using opacity and damage severity confirmed a higher frequency of corneal defects in Akt3^-/+^ mice than in other groups (**Figure 1B**). Together, these data reveal a previously unreported corneal phenotype associated with Akt3 deficiency.

### Loss of Akt3 exerts protection against HSV-1 infection

**Figure 2:**
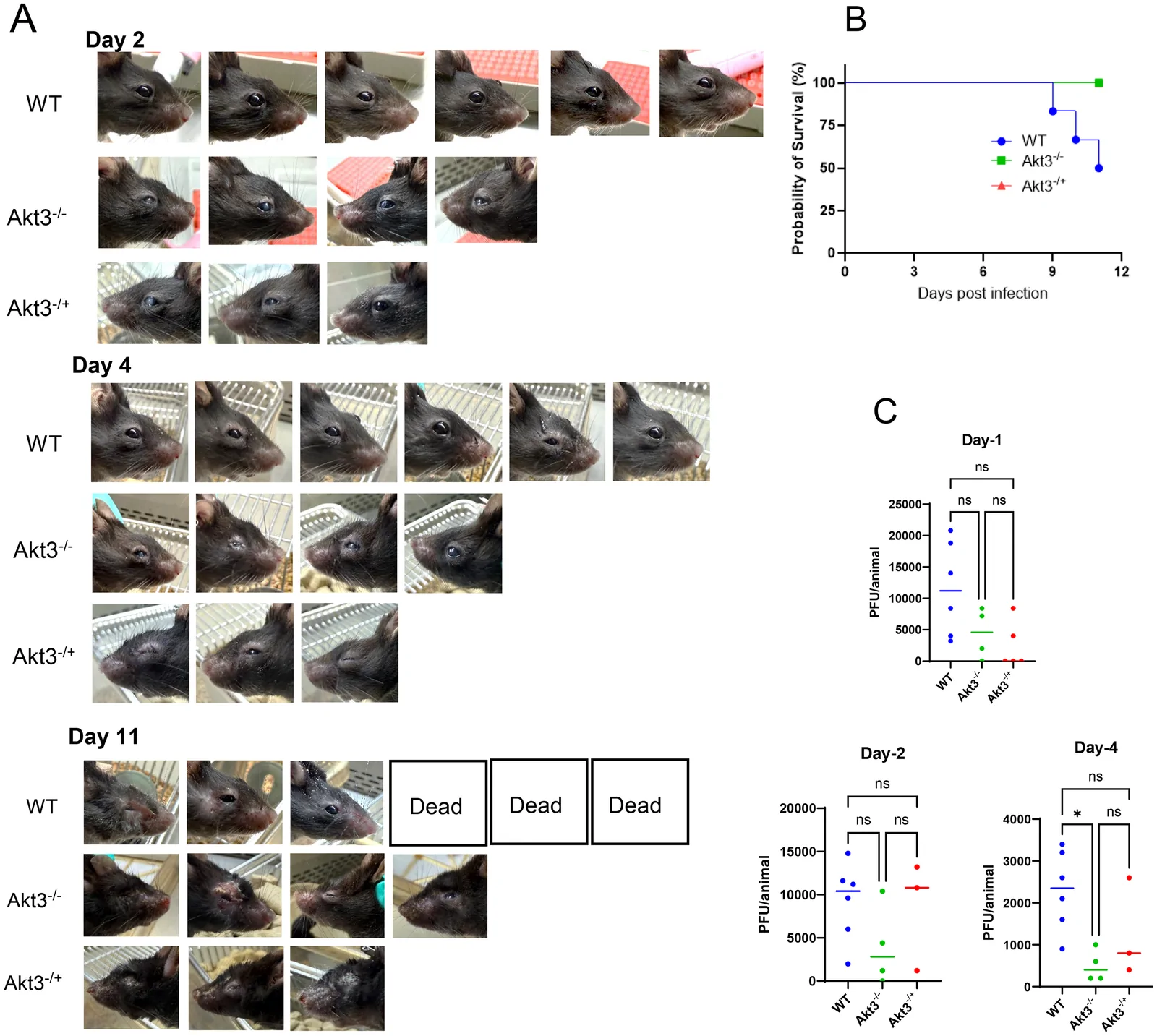
Loss of Akt3 exerts protection against HSV-1 infection. **(A)** Representative cornea images from Akt3^-/-^ and Akt3^-/+^ animals upon HSV-1 infection at Days 2,4, and 11. The images shown correspond to selected eyes from the total number of animals analyzed in each experimental group and are intended to illustrate the range of observed disease phenotypes. The number of images shown (WT, n=6; Akt3^-/-^, n=4; Akt3^-/+^, n=3) indicates the total number of animals included in the analysis for each group. **(B)** Survival curve showing survival probabilities among different groups over time. **(C)** Scatter dot plot indicating virus titer on days 1,2, and 4. For all experiments showing significance, statistical analysis was performed using One-way ANOVA, with significance defined as ns p> 0.05 and ∗p ≤ 0.05.

We next investigated whether baseline corneal opacity in Akt3^-/-^ mice correlated with increased susceptibility to viral infection. Given the observed developmental defects, we hypothesized that Akt3 deficiency would compromise corneal integrity and exacerbate HSV-1 infection. To test this, we challenged WT, Akt3^-/+^ and Akt3^-/-^ mice with HSV-1 corneal scarification (targeting the non-damaged corneas). Unexpectedly, WT animals exhibited progressive virus-induced clinical worsening compared with Akt3-deficient groups (**Figure 2A**). While all Akt3^-/+^ and Akt3^-/-^ mice survived, 50% of the WT group succumbed to HSV-1 infection on day 11 (**Figure 2B**). Viral shedding in eye washes further supported this trend: at 4 days post-infection (dpi), WT mice had significantly higher plaque titers than Akt3^-/-^ mice (**Figure 2C**). Akt3^-/+^ mice showed a similar reduction in viral load, though it did not reach statistical significance. These findings suggest that despite developmental corneal defects, Akt3 deficiency may confer some protection against HSV-1 infection.

### Protective immune responses curb HSV-1 viral infection in Akt3-deficient mice

**Figure 3:**
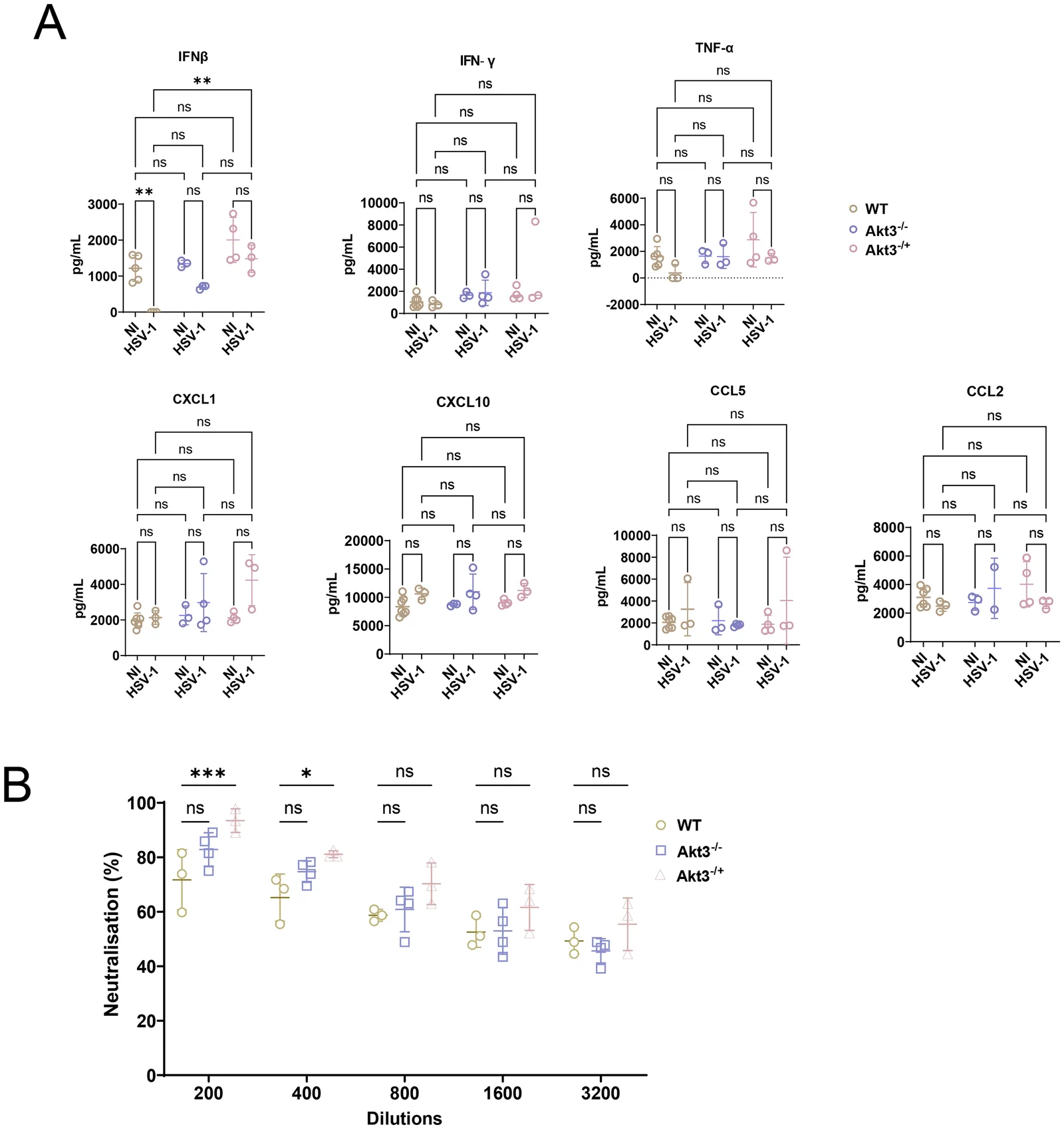
Protective immune responses curb HSV-1 viral infection in Akt3-deficient mice. **(A)** Scatter dot plots showing serum cytokine concentrations in WT, Akt3^+/-^, and Akt3^-/-^ mice at 14 days post-infection (dpi). Each dot represents an individual mouse, and horizontal bars indicate the mean ± SEM. **(B)** Serum neutralizing antibody responses against HSV-1 measured by viral neutralization assay at the indicated serum dilutions at 14 dpi. Neutralization capacity is expressed as the percentage of viral neutralization relative to the virus-only control. Each dot represents an individual mouse, and horizontal bars indicate the mean ± SEM. Statistical significance was determined using one-way ANOVA followed by Tukey’s multiple-comparison test. ns p> 0.05, ∗p ≤ 0.05, ∗∗p ≤ 0.01, ∗∗∗p ≤ 0.001.

We hypothesized that the protection observed in Akt3-deficient mice might stem from a heightened proinflammatory milieu. While we found no significant differences in systemic levels of key chemokines (CXCL1, CXCL10, CCL5, CCL2) or cytokines (TNFα and IFNγ), IFNβ levels were significantly elevated in the Akt3^-/+^ group compared to WT (**Figure 3A**). Interestingly, Akt3^-/-^ mice exhibited the lowest viral titers despite lacking a corresponding increase in IFNβ, suggesting alternative protective mechanisms in the complete knockout. To investigate this, we assessed the HSV-1 neutralizing capacity of the sera. Notably, both Akt3^-/+^ and Akt3^-/-^ mice had significantly higher neutralization titers than WT controls (**Figure 3B**). Our findings indicate that Akt3 deficiency enhances antiviral immunity through a combination of increased IFNβ (in heterozygotes) and augmented humoral responses, suggesting a new role for Akt3 in modulating B cell function during HSV-1 infection.

### Deep immunophenotyping reveals enhanced neutrophil against HSV-1 in Akt3-deficient mice

**Figure 4:**
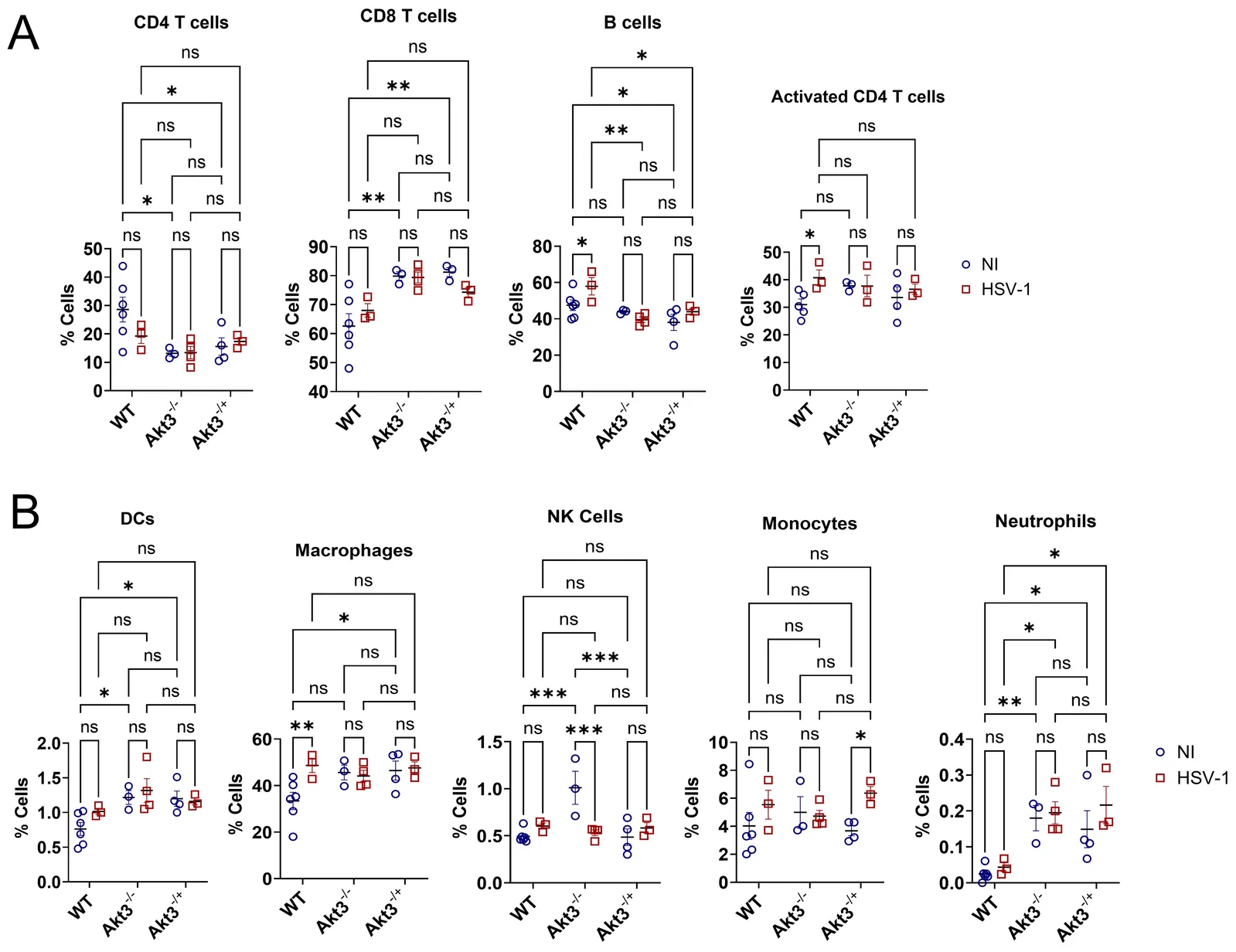
Deep immunophenotyping reveals enhanced neutrophil involvement against HSV-1 in Akt3-deficient mice. **(A, B)** Scatter dot plots showing the frequencies of innate and adaptive immune cell populations in the cervical lymph nodes (cLNs) of non-infected (NI) and HSV-1-infected mice at 14 days post infection. Each dot represents an individual mouse, and horizontal bars indicate the mean ± SEM. Sample sizes were as follows: WT-NI (*n* = 6), WT-HSV-1 (*n* = 3), Akt3^-/-^ -NI (*n* = 3), Akt3^-/-^ -HSV-1 (*n* = 4), Akt3^+/-^-NI (*n* = 4), and Akt3^+/-^-HSV-1(*n* = 3). Statistical analyses were performed using two-way ANOVA. Significance is indicated as ns p> 0.05, ∗p ≤ 0.05, ∗∗p ≤ 0.01, and ∗∗∗p ≤ 0.001.

To delineate the cellular drivers of this antiviral response, we characterized leukocyte subsets in the draining cLNs at 14 days post-infection. While adaptive immune cell populations (B and T cells) showed no significant differences between the genotypes (**Figure 4A**), innate immune profiling revealed a striking phenotype: both Akt3^-/+^ and Akt3^-/-^ mice exhibited a significant expansion of neutrophils compared to WT counterparts (**Figure 4B**). Other innate subsets, including NK cells and macrophages, remained comparable across all three groups. Interestingly, the basal dendritic cell population was higher in both Akt3^-/+^ and Akt3^-/-^ mice prior to infection. Given the role of neutrophils as rapid responders to HSV-1 infection, their enrichment, coupled with elevated IFNβ in Akt3^-/+^ mice, likely primes an enhanced antiviral state. Our findings, therefore, suggest that an augmented neutrophil response in Akt3-deficient mice may provide early viral control while potentially signaling to the humoral arm to enhance the neutralizing antibody responses observed earlier.

## Discussion

In this study, we uncover a new role for Akt3 in the cornea that links tissue integrity with antiviral defense. We observe that loss of Akt3 disrupts corneal clarity, leading to spontaneous opacity, with a more pronounced phenotype in heterozygous animals. Interestingly, this baseline defect does not translate into increased susceptibility to infection. Instead, Akt3-deficient mice show improved control of HSV-1, with lower viral burden and better survival compared to wild-type animals.

This loss of corneal clarity suggests a role for Akt signaling in maintaining corneal homeostasis. Indeed, the corneal epithelium relies on tightly regulated signaling networks to maintain transparency and barrier integrity, with the PI3K/AKT pathway playing a central role in coordinating epithelial survival, proliferation, migration, and redox homeostasis (10). Activation of this pathway by growth factors such as EGF, IGF-1, insulin, and TGF-β supports tissue maintenance and wound healing under physiological conditions (11). Disruption of AKT signaling is therefore expected to impair corneal homeostasis, potentially leading to structural abnormalities such as opacity. This non-linear genotype-phenotype relationship may reflect compensatory mechanisms in the complete knockout, whereas heterozygous mice may retain partial signaling, leading to dysregulated or maladaptive responses during infection. The baseline corneal differences associated with Akt3 dosage may also influence disease scoring and contribute to this pattern. Additional studies will be required to define the underlying mechanism.

Specifically, emerging evidence supports isoform-specific roles of AKT signaling in tissue homeostasis, with Akt3 functioning as a key regulator of cellular integrity across multiple biological systems. Genetic studies have demonstrated that loss of Akt3 leads to reduced brain size due to decreased cell number and size, highlighting its role in regulating tissue growth and structural organization (1, 10, 12). Beyond developmental regulation, Akt3 has been shown to promote cell survival, particularly in stem and progenitor populations, where its loss activates pro-apoptotic pathways, including p53 signaling, leading to increased apoptosis and impaired cell cycle progression (8, 13). In addition, Akt3 contributes to tissue repair and regeneration by regulating cell migration, proliferation, and extracellular matrix remodeling, and its deficiency is associated with delayed wound healing and disrupted collagen organization (14). Collectively, these findings establish Akt3 as a nonredundant regulator of tissue homeostasis. Consistent with this, the baseline corneal opacity observed in Akt3-deficient mice suggests compromised homeostatic signaling and unveils a previously unrecognized role for Akt3 in maintaining corneal integrity and transparency. In addition to intrinsic defects in epithelial homeostasis, the corneal opacity observed in Akt3-deficient mice may also reflect an altered inflammatory environment. Corneal opacity in the context of HSV-1 infection is frequently driven by immune cell infiltration, cytokine production, and tissue remodeling (7, 15, 16). The increased neutrophil frequency and enhanced antiviral immune responses observed in Akt3-deficient mice suggest that heightened innate immune activation may contribute to both improved viral control and tissue pathology. In this context, Akt3 may function not only as a regulator of epithelial integrity but also as a modulator of inflammatory responses within the cornea.

In line with this, HSV-1 actively engages and sustains AKT signaling to establish a permissive intracellular environment for infection. HSV-1 induces and maintains Akt phosphorylation to support viral protein synthesis, cellular survival, and the metabolic activity required for efficient replication.

In this study, our findings that Akt3 deficiency reduces viral burden are consistent with a model in which disruption of Akt signaling compromises viral replication. One possible explanation is that loss of Akt3 could reflect increased susceptibility of infected cells to premature cell death, thereby limiting the time available for productive viral replication. Importantly, our data extend these observations by identifying Akt3 as an isoform-specific contributor to this pro-viral signaling axis, suggesting that individual AKT isoforms may differentially regulate host-virus interactions. Moreover, beyond its role in supporting viral permissiveness, Akt3 appears to modulate host antiviral immunity, indicating that AKT signaling not only facilitates viral replication but may also restrain protective immune responses during ocular HSV-1 infection. Thus, the enhanced antiviral immune responses observed in Akt3-deficient mice suggest that both cell-intrinsic and immune-mediated mechanisms contribute to viral control.

Our data support a context-dependent role for AKT signaling in the cornea, where it is essential for tissue integrity yet is co-opted by HSV-1 to promote infection. In this dual functional model, loss of Akt3 may impair corneal integrity while simultaneously limiting HSV-1 infection. Systemically, the cytokine data reveal a more complex global shift in inflammation. While most measured inflammatory mediators remained unchanged post-infection, IFNβ was selectively increased in infected Akt3^-/+^ mice, suggesting that type I interferon signaling contributes to an anti-HSV-1 environment. Type I interferons are central to resistance against ocular HSV-1 infection, limiting local viral spread and neuro-invasion. Dysregulation in type I IFN signaling increases susceptibility to viral invasion, whereas IFNβ can exert potent anti-HSV activity (9, 17, 18).

The elevated IFNβ observed in heterozygous animals is consistent with a more antiviral immune state; however, other factors may be at play. In our study, the Akt3^-/-^ mice showed the lowest viral titers without a corresponding increase in circulating IFNβ, suggesting possible compensatory mechanisms. These distinct findings between the two Akt3-deficient backgrounds are important because they suggest that Akt3-dependent antiviral regulation may be multifactorial and genotype-dependent, with heterozygous and homozygous deficiencies determining the interactome that restricts HSV-1. We acknowledge that the observed phenotype likely reflects the combined effects of multiple host factors, including antibody-mediated neutralization, innate immune responses, and changes in inflammatory cell dynamics, rather than IFNβ alone.

Furthermore, our immunophenotyping data reveal a selective expansion of neutrophils in the draining lymph nodes of Akt3-deficient mice, suggesting that loss of Akt3 preferentially alters innate immune dynamics. While adaptive immune cell frequencies remained unchanged, neutrophil enrichment suggests a potential upstream regulatory role for Akt3 in innate responses that influence downstream immunity. Neutrophils are well established as rapid responders during HSV-1 infection and contribute to initial viral containment; however, their role extends beyond direct antiviral activity (19). Emerging evidence indicates that neutrophils can modulate germinal center reactions, promote B cell activation, and enhance antibody responses through cytokine production and cellular interactions (20–22). Additionally, genotype-dependent differences in baseline DC populations may also influence the magnitude and quality of both innate and adaptive immune responses following HSV-1 infection. As observed in our study, the pre-existing increase in DCs could also contribute to enhanced protection against HSV-1 infection and increased neutralizing antibody responses.

In this context, the increased neutrophil presence observed in Akt3-deficient mice may create a microenvironment that favors enhanced humoral immunity, consistent with the elevated neutralizing antibody responses identified in these animals (23–26). Although the present study does not directly establish a causal link between neutrophils and B cell function, these findings support a model in which Akt3 acts as a regulator of innate-adaptive crosstalk, where its loss promotes an immune landscape that enhances antiviral protection.

In conclusion, our findings support established evidence that HSV-1 modulates AKT signaling to promote infection, while also revealing a novel isoform- and genotype-specific role for Akt3 in modulating innate immune responses, with potential downstream effects on humoral immunity.

## Data Availability

The raw data supporting the conclusions of this article will be made available by the authors, without undue reservation.
